# 3,4-Dihydroxyphenylacetate 2,3-dioxygenase from *Pseudomonas aeruginosa*: An Fe(II)-containing enzyme with fast turnover

**DOI:** 10.1371/journal.pone.0171135

**Published:** 2017-02-03

**Authors:** Soraya Pornsuwan, Somchart Maenpuen, Philaiwarong Kamutira, Pratchaya Watthaisong, Kittisak Thotsaporn, Chanakan Tongsook, Maneerat Juttulapa, Sarayut Nijvipakul, Pimchai Chaiyen

**Affiliations:** 1 Department of Chemistry, Faculty of Science, Mahidol University, Bangkok, Thailand; 2 Department of Biochemistry, Faculty of Science, Burapha University, Chonburi, Thailand; 3 Department of Biochemistry and Center for Excellence in Protein and Enzyme Technology, Faculty of Science, Mahidol University, Bangkok, Thailand; 4 Department of Biochemistry, Faculty of Dentistry, Chulalongkorn University, Bangkok, Thailand; National Research Council, ITALY

## Abstract

3,4-dihydroxyphenylacetate (DHPA) dioxygenase (DHPAO) from *Pseudomonas aeruginosa* (PaDHPAO) was overexpressed in *Escherichia coli* and purified to homogeneity. As the enzyme lost activity over time, a protocol to reactivate and conserve PaDHPAO activity has been developed. Addition of Fe(II), DTT and ascorbic acid or ROS scavenging enzymes (catalase or superoxide dismutase) was required to preserve enzyme stability. Metal content and activity analyses indicated that PaDHPAO uses Fe(II) as a metal cofactor. NMR analysis of the reaction product indicated that PaDHPAO catalyzes the 2,3-extradiol ring-cleavage of DHPA to form 5-carboxymethyl-2-hydroxymuconate semialdehyde (CHMS) which has a molar absorptivity of 32.23 mM^-1^cm^-1^ at 380 nm and pH 7.5. Steady-state kinetics under air-saturated conditions at 25°C and pH 7.5 showed a *K*_m_ for DHPA of 58 ± 8 μM and a *k*_cat_ of 64 s^-1^, indicating that the turnover of PaDHPAO is relatively fast compared to other DHPAOs. The pH-rate profile of the PaDHPAO reaction shows a bell-shaped plot that exhibits a maximum activity at pH 7.5 with two p*K*_a_ values of 6.5 ± 0.1 and 8.9 ± 0.1. Study of the effect of temperature on PaDHPAO activity indicated that the enzyme activity increases as temperature increases up to 55°C. The Arrhenius plot of ln(*k’*_cat_) *versu*s the reciprocal of the absolute temperature shows two correlations with a transition temperature at 35°C. Two activation energy values (*E*_a_) above and below the transition temperature were calculated as 42 and 14 kJ/mol, respectively. The data imply that the rate determining steps of the PaDHPAO reaction at temperatures above and below 35°C may be different. Sequence similarity network analysis indicated that PaDHPAO belongs to the enzyme clusters that are largely unexplored. As PaDHPAO has a high turnover number compared to most of the enzymes previously reported, understanding its biochemical and biophysical properties should be useful for future applications in biotechnology.

## Introduction

The biodegradation of aromatic compounds is essential for recycling carbon sources in nature. Lignin, one of the most abundant biomasses found in nature is composed of aromatic compounds in the forms of benzenoid and phenolic derivatives [1]. Enzymes involved in the process of lignin degradation are of interest in biorefinery for the conversion of low value biomass into valuable chemicals [2–8]. The biodegradation of phenolic acids is often initiated with *ortho*-hydroxylation to form catecholic compounds by flavin-dependent or metal-dependent hydroxylases [9–11]. The resulting catecholic derivatives are generally further converted into ring-cleaved products by dioxygenases that catalyze C-C bond breaking either adjacent to the vicinal OH substituents (‘extradiol’ cleavage) or between the two hydroxyl groups (‘intradiol’ cleavage) [12, 13]. The breakage of catecholic compounds is a key step in the conversion of stable aromatic derivatives into aliphatic acids that can be further assimilated by microbes. For biotechnology applications, pathways involving the oxidative degradation of aromatic compounds are valuable for the microbial production of useful chemicals such as succinic or lactic acid [7, 14].

**Fig 1:**
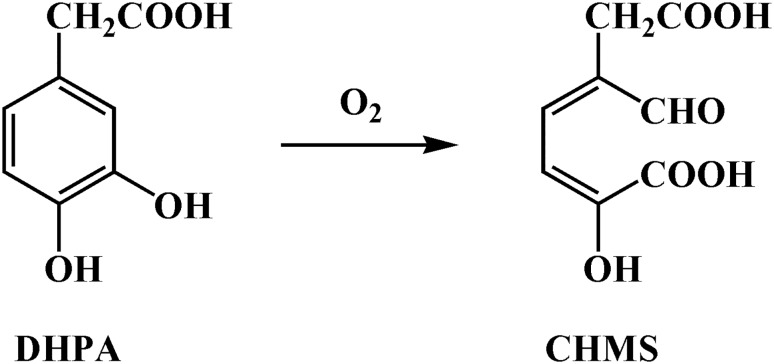
An extradiol cleavage of DHPA by DHPAO.

3,4-Dihydroxyphenylacetate 2,3-dioxygenase (DHPAO) or homoprotocatechuate 2,3 dioxygenase (HPCD) is a mononuclear non-heme metal-containing enzyme that catalyzes the extradiol ring cleavage of 3,4-dihydroxyphenylacetate (DHPA) by incorporation of molecular oxygen to yield 5-carboxymethyl-2-hydroxymuconate semialdehyde (CHMS) (Fig 1) [15, 16]. DHPAO from various organisms were isolated, studied and found to have different catalytic properties. The metal cofactors of DHPAOs from different species show significant diversity and include Fe(II), Mn(II) and Mg(II).

A mononuclear non-heme ferrous ion, Fe(II), is the metal cofactor found in the enzymes isolated from *Pseudomonas ovalis* [17–19] and *Brevibacterium fuscum* [20], while the enzymes from *Bacillus brevis* [21], *Arthrobacter* Mn-1 and *Arthrobacter globiformis* CM-2 [22–24] use Mn(II) as a cofactor. Magnesium ion, Mg(II), was uniquely found to be the cofactor for DHPAO from *Klebsiella pneumoniae* [25]. Over the past two decades, extensive and elegant studies have been carried out with the enzymes from *A*. *globiformis* CM-2 and *B*. *fuscum*. These two enzymes share 83% protein sequence identity and belong to the 2-His-1-carboxylate facial triad enzyme superfamily in which the active-site side chains of two histidine and one glutamate and two or three water molecules are coordinated with a mononuclear non-heme metal cofactor [16, 26–28]. Several intermediates of the enzyme from *B*. *fuscum* were captured in X-ray crystal structures. The results indicated that O_2_ insertion occurs through the substrate-alkylperoxo-Fe(II) intermediate [29–32]. Mutation of the second sphere conserved residue, His200 resulted in the enzyme reacting slowly with 4-nitrocatechol (4-NC), allowing the kinetic detection of an Fe-oxygen intermediate. Replacing Fe(II) with Mn(II) in the active site of DHPAO from *B*. *fuscum* had no significant impact on *K*_m_ or *V*_max_ [28], while substitution with cobalt resulted in an enzyme with higher activity than the Fe(II)-containing enzyme [33, 34]. Table 1 summarizes the biochemical and catalytic properties of DHPAO previously reported. The ability of DHPAO to insert O_2_ into DHPA and generate a yellow ring cleavage product, 5-carboxymethyl-2-hydroxymuconate semialdehyde (CHMS), is useful for chromogenic reporter assays. DHPAO has been used as a coupling enzyme for assaying the reaction of *p*-hydroxyphenylacetate (HPA) 3-hydroxylase (HPAH) which hydroxylates HPA to form DHPA as a product [8, 35].

**Table 1:** Summary of biochemical and catalytic properties of DHPAO from various organisms.

Organisms	Metal center (mole per tetramer)	Specific activity (U/mg protein)	Native molecular mass (Da)	Activity increased upon Fe(II) activation	Kinetic parameters			Ref.
					*K*_m,DHPA_ (μM)	*k*_cat_ (s^-1^)	*k*_cat_/*K*_m_ (μM^-1^s^-1^)	
*Pseudomonas ovalis*	Fe(II) (4)	30	140,000 (4x35,000)	Yes	nr.	nr.	nr.	[19]
*Brevibacterium fuscum*	Fe(II) (4)	9–14	220,000 (4x42,500)	Yes	15	10	0.67	[20]^a^
*Bacillus brevis*	Mn(II) (1.9–2.1)	6	140,000 (4x36,000)	No	14	nr.	nr.	[21]^b^
*Arthrobacter globiformis* CM-2	Mn(II) (2.8–3.3)	9	155,444 (4x38,861)	No	7	23.3	3.33	[24]^c^
*Klebsiella pneumoniae*	Mg(II) (1.0–1.3)	102	102,000 (4x25,500)	No	74–77	nr.	nr.	[25]^d^
*Bacillus stearothermophilus*	nr.	0.275	106,000 (3x35,000)	No	3.4 ± 0.2	0.114	0.033	[36]^e^
*Escherichia coli* C	nr.	100	33,000 for monomer	No	16 ± 3	nr.	nr.	[37]^f^

nr., no record

^*a*^pH 7.5, 23°C

^*b*^pH 7.5, 25°C

^*c*^pH 8.0, 23°C

^*d*^pH 7.2–7.8

^*e*^pH 8.2, 32°C

^*f*^pH 7.5, 30°C

In this work, we report on the overexpression and characterization of a new extradiol cleaving dioxygenase, DHPAO from *Pseudomonas aeruginosa* (PaDHPAO). The enzyme sequence is significantly different from other well studied DHPAOs. We have established procedures for enzyme purification, activation and storage. Steady-state kinetic data indicate that the turnover number of this enzyme is rather high compared to other DHPAOs. Our results contribute to the understanding of the diversified properties of extradiol ring-cleaving dioxygenases and identify a novel enzyme that may be useful for biotechnology applications in the future.

## Materials and methods

### Materials

All chemicals were of the highest available purity and purchased from Sigma Aldrich (St. Louis, MO, USA). DEAE- and Phenyl-Sepharose chromatographic media were purchased from GE Healthcare.

### Cloning and sequence analysis of the *dhpao*gene from *Pseudomonas aeruginosa* PAO

The genomic DNA of *Pseudomonas aeruginosa* PAO was used as a template for PCR amplification. The primers, *dhpao*-sense (5’-GAATCCATATGGGCAAAGTCGCTC-3’) and *dhpao*-antisense (5’-GGTGGGATCCTCAGAGGCGGCTG-3’), were designed according to the sequence of the *dhpao* gene from *P*. *aeruginosa* PAO deposited in the NCBI database. *Nde*I and *Bam*HI restriction sites (underlined in the sequences) were incorporated into the *dhpao*-sense and *dhpao*-antisense, respectively. PCR (50 μL total volume) was performed with 50 ng of genomic DNA, 200 μM of dNTP, 1 μM of each primer and 1 unit of *Pfu* DNA polymerase. The amplification condition was as follows: pre-denaturation at 95°C for 10 min and amplification for 30 cycles (denaturation at 95°C for 1 min, annealing at 55°C for 1 min and extension at 72°C for 1 min). After the last cycle, the reaction was extended at 72°C for 10 min. The PCR product was extracted, purified from an agarose gel, digested with *Nde*I and *Bam*HI, and then ligated with an *Nde*I/*Bam*HI digested pET-11a plasmid. The resulting recombinant plasmid was transformed into *E*. *coli* XL1-Blue in LB media containing ampicillin. DNA from a positive clone was sequenced to verify that it indeed contains the *dhpao* gene.

The molecular weight, isoelectric point (pI) and molar absorption coefficient at 280 nm of DHPAO from *P*. *aeruginosa* (PaDHPAO) were calculated using the online tools on the Expasy bioinformatics resource portal (http://web.expasy.org/compute_pi/), and the conserved domain was analyzed by the conserved domain architecture retrieval tool (http://www.ncbi.nlm.nih.gov/Structure/lexington/lexington.cgi) on the NCBI website.

### Enzyme expression and purification

*E*. *coli* BL21 (DE3) cells containing plasmids harboring the *Padhpao* gene, were used for inoculating 3.2 L of LB media containing 50 μg/mL of ampicillin at 37°C. Cells were grown until the OD_600_ of the cell culture reached ~1.0. The shaker temperature was then adjusted to 25°C and isopropyl *β*-D-thiogalactopyranoside (IPTG) was added into the culture to a final concentration of 1 mM to initiate protein expression. The temperature was maintained at 25°C after IPTG addition. The culture was harvested when the OD_600_ reached ~3.0–4.0 using centrifugation at 8,000 rpm for 8 minutes. The cell paste was stored at -80°C until used.

All steps of the enzyme purification were carried out at 4°C and all buffers used for this purification were bubbled with purified nitrogen gas before use. Frozen cell paste (~12 g) was thawed and resuspended in a lysis buffer (50 mM potassium phosphate buffer, pH 7.0, 1 mM DTT and 100 μM PMSF). Cells were disrupted by ultrasonication with 80% amplitude for 20 minutes. The cell lysate was then centrifuged at 15,000 rpm at 4°C for 1 hour. The pellet was discarded and the supernatant was defined as the crude extract. Nucleic acid material was removed from the crude extract by adding a solution of 10%(w/v) of polyethyleneimine (PEI) to achieve a final concentration of 1%(w/v) in the crude extract. The cell suspension was centrifuged at 15,000 rpm at 4°C for 30 minutes and the pellet was discarded. The supernatant was loaded onto a DEAE-Sepharose column equilibrated with 1 L of 50 mM potassium phosphate, pH 7.0 containing 1 mM DTT. The column was washed with 1 L of the same buffer. The enzyme was eluted with a 2 L gradient of 0–300 mM NaCl in 50 mM potassium phosphate, pH 7.0 containing 1 mM DTT. Enzyme fractions were pooled according to absorption at 280 nm, and then concentrated using a stirred cell (Amicon^®^ 8050 or 8200) equipped with a Millipore YM-10 membrane (10 kDa cut-off). The concentrated enzyme was added to a saturated ammonium sulfate solution to yield a final ammonium sulfate concentration of 80%(w/v). The ammonium sulfate suspension was then centrifuged at 15,000 rpm at 4°C for 30 minutes to obtain the enzyme pellet fraction. The pellet was completely dissolved in 50 mM potassium phosphate buffer, pH 7.0 containing 1 mM DTT and was subsequently loaded onto a Phenyl-Sepharose column equilibrated with 15%(w/v) ammonium sulfate in 50 mM potassium phosphate, pH 7.0 containing 1 mM DTT. The column was washed with the same buffer and then eluted with a gradient of 15–0%(w/v) ammonium sulfate in 50 mM potassium phosphate buffer, pH 7.0 containing 1 mM DTT. The enzyme was pooled and concentrated as mentioned above. The concentrated enzyme was passed through a Sephadex G-25 column equilibrated with 30 mM 3-morpholinopropane sulfonate (MOPS) buffer, pH 7.0. The concentration of enzyme was determined using a molar absorption coefficient of 49.06 mM^-1^cm^-1^ at 280 nm. The enzyme solution was then aliquoted and stored frozen at -80°C until used. A specific activity of the purified PaDHPAO was determined according to the enzyme assay method as described in detail below.

### Molecular mass determination of the native PaDHPAO

The molecular mass of PaDHPAO was determined by gel filtration chromatography (Superdex200 analytical grade) operated on an FPLC instrument. The Superdex200 column was equilibrated with 50 mM sodium phosphate buffer, pH 7.0 containing 150 mM NaCl. The eluted protein was collected at a flow rate of 0.5 mL·min^-1^ and the elution volume (V_e_) was used to calculate protein molecular weights. Blue dextran (2000 kDa) was used to determine the void volume (V_o_). Ferritin (440 kDa), aldolase (158 kDa), BSA (65.4 kDa), ovalbumin (48.9 kDa), chymotrypsinogen (22.8 kDa), and ribonuclease (15.8 kDa) were used as protein standards. Determination of molecular mass of the protein components was carried out by comparing the ratio of V_e_ to V_o_ to those of the protein standards. A standard calibration curve was plotted between logarithms of the known molecular mass *versus* their V_e_/V_o_ ratios. To determine the subunit molecular mass of PaDHPAO, sodium dodecylsulfate polyacrylamide gel electrophoresis (SDS-PAGE) was performed on a 12%(w/v) gel at pH 8.8 with low-range molecular weight markers (20–75 kDa) (Enzmart Biotech, Thailand). The protein solution was incubated with 50 mM DTT at 100°C for 5 min before loading onto the gel.

### Enzyme assays

DHPAO catalyzes the conversion of DHPA to form 5-carboxymethyl-2-hydroxymuconate semialdehyde (CHMS), a yellow compound with λ_max_ at 380 nm (ε_380_ = 32.23 mM^-1^cm^-1^ at pH 7.5 (see Results)). A typical assay contained 150–390 μM DHPA and oxygen (260 μM) at air-saturation. Reactions were carried out in 50 mM sodium phosphate buffer, pH 7.5 at 25°C. The reactions were started by adding DHPA, and formation of CHMS was monitored by absorbance at 380 nm. The control reaction in the absence of enzyme was also performed. Enzyme activity was calculated from an initial slope of A_380_ *versus* time. One unit of enzyme activity was defined as the amount of enzyme required to form 1 μmol CHMS per minute at 25°C and pH 7.5. For determination of the enzyme specific activity that was obtained from each purification step, the assay reactions were performed in the presence of 100 μM Fe(NH_4_)_2_(SO_4_)_2_ to compensate for the cofactor loss that was due to ferrous oxidation during the purification process.

### Enzyme activation and preservation

To investigate whether the addition of Fe(II) and other reducing agents could increase the activity of PaDHPAO, a solution of 100 μM PaDHPAO was incubated in 50 mM potassium phosphate buffer, pH 7.0 at 25°C for 1 hour in the presence of the following reagents: (1) 10 mM DTT, (2) 0.5 mM Fe(NH_4_)_2_(SO_4_)_2_, (3) 10 mM DTT and 0.5 mM Fe(NH_4_)_2_(SO_4_)_2_, and (4) 0.5 mM ascorbic acid. After incubation, the activated enzyme was loaded onto a PD-10 desalting column equilibrated with the same potassium phosphate buffer to remove excess activating reagents. The enzyme assay was carried out by adding 5 nM of activated PaDHPAO into a solution of 150 μM DHPA in 50 mM sodium phosphate buffer, pH 7.5. The control reaction in the absence of enzyme was also performed.

Due to the drastic loss of relative activity (35–65% after 2–7 hours) observed for the activated enzyme even while kept on ice (see the result in “Preservation of the activated PaDHPAO activity” section), reducing agents and reactive oxygen species (ROS) scavenging enzymes (catalase and superoxide dismutase) were added into the buffer to determine whether they could preserve the activity of PaDHPAO. A 1-mL solution of 100 μM PaDHPAO with 0.5 mM Fe(NH_4_)_2_(SO_4_)_2_ and 10 mM DTT in 50 mM potassium phosphate buffer, pH 7.0 was incubated at 25°C for 1 hour to allow enzyme activation (this is a suitable condition for enzyme activation as determined by a previous experiment). After the activation process, the activated enzyme solution was then loaded onto a PD-10 desalting column pre-equilibrated with a 50 mM potassium phosphate buffer, pH 7.0 to remove excess metal ion. The eluted enzyme was collected and concentrated, and the concentration of the activated enzyme was then determined using a molar absorption coefficient at 280 nm of 49.06 mM^-1^cm^-1^. A solution of activated enzyme was then added with different components: (1) no additional agents, (2) 0.5 mM Fe(NH_4_)_2_(SO_4_)_2_, (3) 1 mM DTT, (4) 0.5 mM ascorbic acid, (5) 0.5 mM ascorbic acid and 1 mM DTT, (6) 0.5 mM ascorbic acid, 1 mM DTT and 0.5 mM Fe(NH_4_)_2_(SO_4_)_2_, (7) 1 mM DTT and 0.5 mM Fe(NH_4_)_2_(SO_4_)_2_, (8) 0.5 mM ascorbic acid and 0.5 mM Fe(NH_4_)_2_(SO_4_)_2_, (9) 1%(w/v) catalase, (10) 50 U/mL superoxide dismutase or (11) 1%(w/v) catalase and 50 U/mL superoxide dismutase. Each treated enzyme solution was collected and kept on ice. Their activities were measured immediately and then every hour for up to 7–8 hours. The reaction assay containing 40 nM PaDHPAO and 390 μM DHPA was performed in air-saturated 50 mM sodium phosphate buffer, pH 7.5 at 25°C and the CHMS product formed was monitored by absorbance at 380 nm. The relative activities of PaDHPAO under each condition and after different storage periods were compared to identify the conditions most suitable for maintaining the activated enzyme activity.

### Effect of metal ions and redox active reagents on PaDHPAO activity

In order to examine whether the addition of metal ions and redox active reagents could affect PaDHAPO activity, a solution of 2 μM PaDHPAO in 50 mM potassium phosphate buffer, pH 7.0 was incubated with different reagents at 4°C for 10 minutes and 3.5 hours. After incubation, the enzyme activity was measured as described earlier. Enzyme inactivation was measured as the percentage of activity remaining relative to a control assay with untreated PaDHPAO.

### Metal cofactor specificity

As previous reports showed that an Fe(II)-dependent DHPAO from *B*. *fuscum* can use Mn(II) [28] and Co(II) as cofactors [33], we thus explored whether PaDHPAO can also use other metals besides Fe(II) as cofactors. To perform this experiment, apoenzyme of PaDHPAO (apoPaDHPAO) was prepared by incubating PaDHPAO (618 μM) with about 8-fold excess EDTA (5 mM) on ice overnight. After the incubation, the mixture was loaded onto a Sephadex G-25 column (1.5 × 15.0 cm; volume ~38 mL) equilibrated with 50 mM potassium phosphate buffer, pH 7.0. The eluted enzyme was then collected and concentrated. The concentration of apoPaDHPAO was determined using a molar absorption coefficient at 280 nm of 49.06 mM^-1^cm^-1^. The apoPaDHPAO was reconstituted with Fe(II), Mn(II) or Co(II) by incubating 100 μM apoPaDHPAO (1 mL each) with 0.5 mM of the metal ion (Fe(NH_4_)_2_(SO_4_)_2_, MnCl_2_, or CoCl_2_) in 50 mM potassium phosphate buffer, pH 7.0 and 10 mM DTT at 25°C for 1 hour. The enzyme solution was then loaded onto a PD-10 desalting column equilibrated with 50 mM potassium phosphate buffer, pH 7.0 to remove excess metal ions. The activity of each metal-added enzyme was measured according to the enzyme assay protocol as mentioned earlier (40 nM PaDHPAO and 390 μM DHPA in air-saturated 50 mM sodium phosphate buffer, pH 7.5 at 25°C) and then compared to the activity of apoPaDHPAO and active PaDHPAO.

To investigate whether each metal ion candidate can bind to the enzyme, the dye-binding thermal shift assay of each metal-added enzyme was carried out using a real-time PCR machine [38, 39]. A solution (20 μM) of apoPaDHPAO and apoPaDHPAO reconstituted with each metal candidate (Fe, Mn, and Co) was mixed with an appropriate concentration (5X) of a SYPRO Orange dye solution (Invitrogen, USA). The fluorescence of each sample was monitored using a real-time PCR machine with a temperature increase from 25 to 95°C with a constant increment of 1°C/min. The melting temperature (T_m_) of each PaDHPAO sample was analyzed for a temperature in which the protein is 50% unfolded [40].

### Metal content analysis

Analysis of metal content was first carried out by inductively coupled plasma-mass spectroscopy (ICP-MS) to identify the metal species bound to the purified enzyme. Three metal ions –Fe, Mn, and Zn– were found at significant levels (data not shown). However, as results in the previous section (see the result in “Metal specificity and analysis” section) indicated that only Fe(II) can serve as a cofactor of PaDHPAO, the binding of Fe(II) to PaDHPAO was further investigated using inductively coupled plasma-optical emission spectrometry (ICP-OES) (Perkin-Elmer Optima 7300 DV) and microwave plasma-atomic emission spectroscopy (MP-AES) (Agilent Technologies). The samples for metal analysis were apoPaDHPAO and Fe(II)-reconstituted apo-enzymes. To prepare the Fe(II)-reconstituted apo-enzyme, a 1-mL solution of 100 μM apoPaDHPAO in 10 mM MOPS buffer, pH 7.0 was added with 0.5 mM Fe(NH_4_)_2_(SO_4_)_2_ and 10 mM DTT and the solution was incubated for an hour at 25°C. The Fe(II)-reconstituted apoPaDHPAO was then loaded onto a PD-10 desalting column pre-equilibrated with 10 mM MOPS buffer, pH 7.0 to remove the excess Fe(II) and DTT. The holoenzyme was collected, and the exact protein concentration was determined. The concentration of Fe in each sample was determined using a calibration curve of Fe (1000 ppm stock in 2% HNO_3_) (Perkin-Elmer) at concentration ranges of 0.025 to 2 ppm for ICP-OES analysis, respectively. The buffer, 10 mM MOPS, pH 7.0 was used as a blank. The data were used for calculating the mole ratio of metal:enzyme.

### Product identification

^1^H-NMR was used to identify the product obtained from the DHPAO ring-cleavage reaction. The product was generated by adding 5 μM PaDHPAO into 40 mL of 10 mM ammonium bicarbonate buffer, pH 7.5 containing 250 μM DHPA. The reaction was incubated at room temperature and product formation was monitored by an increase in absorbance at 380 nm (A_380_). The reaction was allowed to proceed until the A_380_ remained constant, which occurred within 10–20 min. The enzyme was then removed by ultrafiltration with a 10 kDa cut-off Centriprep at 4°C. The filtrate was lyophilized and then resuspended in *d*_*6*_-DMSO for ^1^H-NMR measurements (Bruker AVANCE 500 MHz).

### Effect of pH on product absorptivity

To evaluate the effect of pH on the product absorption properties, the product was prepared according to the procedure used for the ^1^H-NMR measurements, and the solution of product with a final concentration of 30 μM was added into a 1-cm quartz cuvette containing buffers at various pHs (pH 4.5–12.0). The buffers used in this experiment to maintain various pH values were 100 mM acetate/acetic acid (pH 4.5–5.5), 100 mM sodium phosphate (pH 6.0–7.5), 100 mM Tris-HCl (pH 8.0–8.5), and 100 mM glycine adjusted with NaOH (pH 9.0–10.0) and 100 mM sodium phosphate (pH 10.5–12.0). The absorbance spectra of the product at pH 4.5–12.0 were monitored between 200–600 nm. The results showed that the absorbance spectra of the ring-cleavage product, CHMS, have a clear isosbestic point at 350 nm with an extinction coefficient of 13.3 mM^-1^cm^-1^.

### Substrate specificity

Enzyme activities with various substrates were explored by adding PaDHPAO (final concentration of 0.02–2 μM) to 50 mM sodium phosphate buffer, pH 7.5 containing 0.5 mM of various catecholic compounds. In the case of slow substrates such as 3,4-dihydroxybenzoate, 4-hydroxyphenylacetate, 4-hydroxybenzoate, 4-hydroxyphenylpropionate, 4-hydroxy-3-methoxyphenylacetate, and 4-nitrocatecol, the final concentration of PaDHPAO used was 2 μM. The appearance of ring-cleavage products was monitored at the wavelength which gives the highest absorption coefficient [19, 24]. The relative activity was calculated by comparing the observed initial rates of the reactions with substrate analogues to the reaction with the native substrate, DHPA.

### Steady-state kinetics measurement

To monitor the PaDHPAO reaction at various DHPA concentrations, the initial velocities of the reactions were carried out at 25°C in air-saturated (oxygen concentration ~260 μM) 50 mM sodium phosphate buffer, pH 7.5 using spectrophotometry. To prepare the activated enzyme, 0.5 mM (Fe(NH_4_)_2_(SO_4_)_2_ and 10 mM DTT was added to a 1-mL solution of 100 μM PaDHPAO and incubated for 1 hour at 25°C. The activated enzyme solution was then passed through a PD-10 desalting column equilibrated with 50 mM potassium phosphate buffer, pH 7.0 to remove excess metal ions and other small molecules. The activated enzyme was then diluted in the same buffer with or without 0.5 mM ascorbic acid to prepare a working stock enzyme solution. Steady-state kinetic assays for the activated PaDHPAO (39 nM) that was prepared in a buffer with or without ascorbic acid were performed in a 1-mL reaction of 50 mM sodium phosphate buffer, pH 7.5 containing various concentrations of DHPA (0.5–2560 μM). The reaction was monitored at 380 nm using the absorption coefficient value of 32.23 mM^-1^cm^-1^. The initial velocity (*v*_o_) data obtained from different enzyme preparations were analyzed as a function of DHPA concentrations to determine the maximum velocity (*V*_max_). The Michaelis constants (*K*_m_) were calculated by fitting the data to the Michaelis-Menten equation. The data were analyzed using Marquardt-Lavenberg algorithms in the KaleidaGraph version 4.0 software (Synergy Software). The steady-state kinetic parameters of PaDHPAO prepared in a buffer either with or without ascorbic acid were compared.

### Effects of pH and temperature on the PaDHPAO reaction

To determine the optimum pH for the PaDHPAO reaction, the assays were carried out at 25°C in 100 mM buffer at various pH conditions (4.0–10.5) and monitored at 350 nm (the isosbestic point) to avoid any influence of pH on the product absorbance. The buffers used in this experiment were sodium acetate for pH 4.0–5.5, sodium phosphate for pH 6.0–7.5, Tris-HCl for pH 8.0 and 8.5, and glycine-NaOH for pH 9.0–10.5. The assays were performed in a 1-mL reaction containing 390 μM DHPA and 39 nM PaDHPAO. The control reaction under each pH condition in the absence of enzyme was also performed. The initial velocities (*v*_o_) were measured and are represented as the apparent maximum velocities (*V’*_max_). A plot of initial velocities as a function of pH was analyzed according to eq (1) where Y is the apparent maximum velocity (*V’*_max_), C is the pH-independent value of *V’*_max_, and *K*_1_ and *K*_2_ are the dissociation constants for the ionizable groups.

$$Y=\frac{C}{1+\frac{10^{-pH}}{10^{-pK_{1}}}+\frac{10^{-pK_{2}}}{10^{-pH}}}$$ (1)

To investigate the effects of temperature on PaDHPAO, reactions containing 42 nM PaDHPAO and 400 μM DHPA in 1-mL of 50 mM sodium phosphate buffer, pH 7.5 were performed at various temperatures (5–70°C) and the initial velocities were monitored at 380 nm. The initial velocity at saturating substrate concentrations was measured to calculate the apparent catalytic rate constant (*k*’_cat_) for each temperature condition. An Arrhenius plot of ln(*k’*_cat_) *versus* the reciprocal of the absolute temperature (1/T) was analyzed according to the Arrhenius eq (2) to obtain the activation energy (*E*_a_) from the slope (*E*_a_/*R*) of the Arrhenius plot. *R* in the Arrhenius plot is a gas constant (8.314 J/mol/K), T is the absolute temperature and A is the Arrhenius constant. Eqs (1) and (2) were analyzed using Marquardt-Lavenberg algorithms in the KaleidaGraph version 4.0 software (Synergy Software).

$$\text{ln}\left(k_{\text{cat}}^{\text{'}}\right)=\text{ln}\left(A\right)-\frac{E_{\text{a}}}{R\text{T}}$$ (2)

To examine the thermal stability of PaDHPAO under different conditions (apoPaDHPAO, activated PaDHPAO, and activated PaDHPAO plus ascorbic acid), a dye-binding thermal shift assay was performed using a real-time PCR machine [38, 39] as previously described. The experiments were performed in a similar fashion, except that a final concentration of 10 μM of each PaDHPAO sample (apoPaDHPAO, activated PaDHPAO, and activated PaDHPAO plus ascorbic acid) was used instead. The T_m_ of each PaDHPAO sample was analyzed and compared.

### Generation of sequence similarity networks

Sequence similarity networks (SSNs) for PaDHPAO were generated with Option A of the EFI-Enzyme Similarity Tool (EFI-EST; http://efi.igb.illinois.edu/efi-est/) which uses the amino acid sequence of PaDHPAO as the query for a BLAST search of the UniProtKB database. The SSNs were generated from the top 1195 sequences homologous to PaDHPAO identified from blast hits using a minimum E-value of 1 (equivalent to the identity value of ~34% or higher). Networks were constructed based on an alignment score threshold of 60 from the EFI program. This value arranges enzymes which have a sequence identity of ~34% or greater to be in the same cluster. Cytoscape v.3.2.1 [41] was used for visualization and analysis of the SSN. A multiple sequence alignment was also performed using a Clustal Omega program of the EMBL-EBI tool (https://www.ebi.ac.uk/Tools/msa/clustalo/) to compare the amino acid sequence of PaDHPAO with other well-studied DHPAOs and extradiol ring-cleavage dioxygenases and to identify possible active-site residues that could be involved in metal coordination and enzyme catalysis. These enzyme sequences were also analyzed by a pairwise sequence alignment using an EMBOSS Needle program of the EMBL-EBI tool (https://www.ebi.ac.uk/Tools/psa/emboss_needle/).

## Results and discussion

### Sequence analysis of PaDHPAO

**Fig 2:**
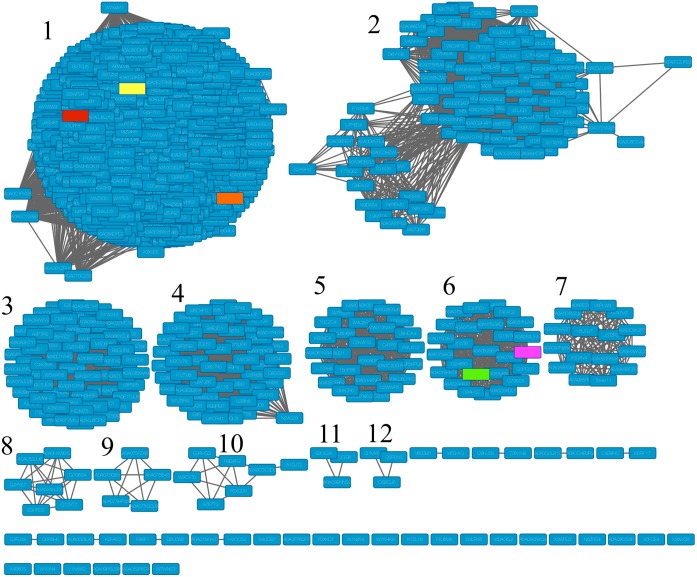
Sequence similarity networks (SSN) for PaDHPAO constructed by EFI alignment score of 60 (~34% identity) illustrating 12 iso-function clusters of enzymes. Enzymes that were experimentally investigated are highlighted in color. PaDHPAO (this study) is yellow, *E*. *coli* C (Uniprot:Q05353) is orange, *K*. *pneumoniae* (Uniprot:Q9RE15) is red, *Pseudomonas* sp. (Uniprot:O33477) is green, and *C*. *testosteroni* (Uniprot:Q6J1Z6) is pink.

The complete sequence of PaDHPAO is encoded by 924 bp, corresponding to a polypeptide of 307 amino acid residues which is equivalent to a subunit molecular weight of 33.4 kDa. The enzyme pI value was calculated as 5.7. Sequence similarity networks of 1,195 enzymes that are closely related to PaDHPAO (~34% and higher protein identity) were generated using Option A of EFI-EST (Fig 2). The EFI-EST analysis separated the proteins into 12 clusters. Proteins in Cluster 1 contain 832 sequences that show sequence identity of 38% or higher to PaDHPAO. Most of these proteins were annotated as DHPAO. Only two proteins in this cluster, DHPAO from *Escherichia coli* C (Uniprot:Q05353) and *Klebsiella pneumoniae* (Uniprot:Q9RE15) which have the sequence identity of ~54–56% to PaDHPAO, were studied experimentally. For Cluster 2, which contains 109 protein sequences having 43% sequence identity to the other members in the same cluster, most of the proteins were assigned as extradiol ring-cleavage dioxygenases with only one enzyme being previously characterized as L-dihydroxyphenylalanine (L-DOPA) 4,5-dioxygenase. It is likely that this cluster represents dioxygenases that catalyze ring-cleavage between the C4 and C5 positions. Other clusters identified had fewer members in their groups and consisted mostly of unexplored dioxygenases with the exception of two members in Cluster 6, 2-aminophenol 1,6-dioxygenase (APD) from *Pseudomonas* sp. (Uniprot:O33477) and *Comamonas testosteroni* (Uniprot:Q6J1Z6), which were characterized as dioxygenases with ring-cleavage activity between the C1 and C6 positions. This EFI analysis suggests that most of the dioxygenases in Cluster 1 have not been explored experimentally and their functions remain unknown. Therefore, the characterization of PaDHPAO in this report should be useful for understanding the functions of the enzymes in this cluster. It is interesting to note that most of the well-studied DHPAOs including the enzymes from *Brevibacterium fuscum* and *Arthrobacter globiformis* CM-2 have very low sequence identity to PaDHPAO-only 12.9% and 6.4%, respectively. These data imply that the structural and functional features of ring-cleavage dioxygenases are diversified and that PaDHPAO is distantly related to the enzymes from *B*. *fuscum* and *A*. *globiformis* CM-2.

Multiple sequence alignment of DHPAOs in Cluster 1 (PaDHPAO, *K*. *pneumoniae* (Kp) DHPAO, and *E*. *coli* C (Ec) DHPAO) and Cluster 6 (APD from *C*. *testosteroni* (CtAPD)) were carried out to identify residues that may be involved in metal-binding and catalysis (S1 Fig). CtAPD is an extradiol ring-cleavage dioxygenase whose X-ray structure is available and its sequence identity to DHPAOs in Cluster 1 is ~21–25%. Based on the crystal structure of CtAPD (PDB codes: 3VSH and 3VHI), the active-site residues His13, His62, and Glu251 are ligated with a mononuclear non-heme Fe(II) center and Tyr129 and His195 are involved in formation of a second sphere metal coordination (S1 Fig) [42]. Based on the sequence analysis (S1 Fig), the three active-site residues involved in Fe(II) coordination of CtAPD are all conserved in PaDHPAO as well as other DHPAOs in Cluster 1 (His12, His57, and Glu239 of PaDHPAO as highlighted in yellow in S1 Fig). The data suggest that the metal coordination of PaDHPAO and other DHPAOs in Cluster 1 are likely involved with 2-His-1-carboxylate facial triad to coordinate a mononuclear non-heme iron bound at the enzyme active site [43–45]. The sequence analysis also identified Tyr125 and His186 in PaDHPAO and Tyr and His in other DHPAOs in Cluster 1 (highlighted in cyan, S1 Fig) which are homologous to Tyr129 and His195 of CtAPD. These data indicate that the second sphere metal-coordination of PaDHPAO is also similar to that of CtAPD (S1 Fig).

### Expression and purification of PaDHPAO

**Fig 3:**
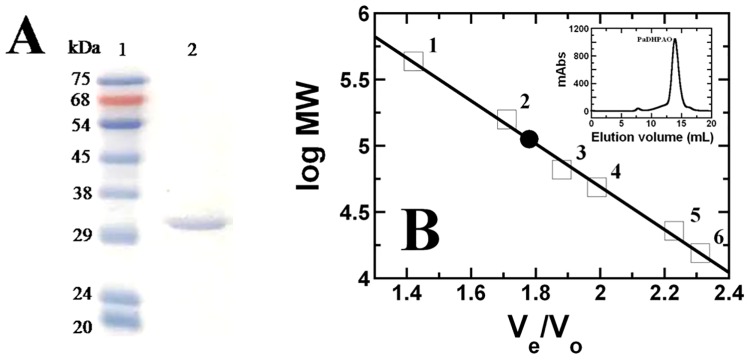
Molecular mass of purified PaDHPAO. (A) SDS-PAGE (12%(w/v)) of the purified PaDHPAO. Lane 1 is a protein molecular weight standard marker (kDa) (Enzmart Biotech, Thailand) and lane 2 is an enzyme solution after purification by Phenyl-Sepharose chromatography. (B) A plot of relative volumes (V_e_/V_o_) *versus* the logarithms of the known molecular masses of protein standards (□): (1) ferritin (440 kDa), (2) aldolase (158 kDa), (3) BSA (65.4 kDa), (4) ovalbumin (48.9 kDa), (5) chymotrypsinogen (22.8 kDa), (6) ribonuclease (15.8 kDa), and PaDHPAO (●).

The protocol reported in the Materials and Methods typically resulted in ~221 mg of purified PaDHPAO per 3.2 L of cell culture (12 g of wet cell paste). The efficiency of each purification step is summarized in Table 2. SDS-PAGE analysis of the purified enzyme indicates a subunit molecular weight of approximately 30 kDa with >95% protein purity as shown in Fig 3A. A native molecular mass of 112.2 kDa was determined based on gel filtration chromatography (Fig 3B). The results of the subunit and native molecular mass determination are consistent with the enzyme being a homotetramer (4×30 kDa = 120 kDa). This homotetrameric structure is also similar to the quaternary structures of other known DHPAOs (see Table 1). Due to the loss of cofactor during purification, the specific activity measured at each purification step was determined in the presence of 100 μM Fe(NH_4_)_2_(SO_4_)_2_. The purified PaDHPAO had a specific activity of 44.3 U/mg protein with a recovery yield of 45.9%. The activation of extradiol catechol dioxygenase by iron (II) and a reducing agent (ascorbic acid) has also been observed in 2,3-dihydroxyphenylpropionate 1,2-dioxygenase (MhpB) from *E*. *coli* [46].

**Table 2:** Summary of PaDHPAO purification.

Sample	Total volume (mL)	Total protein (mg)	Total activity (U)	Specific activity (U/mg)	%Yield	Purification fold
Crude extract	20	1,192	21,300	17.9	100	1
1%PEI	16	796.8	12,800	16.1	60.6	0.9
DEAE-Sepharose	18.5	292.3	13,227	45.3	61.5	2.5
Phenyl-Sepharose	8.9	220.7	9,768	44.3	45.9	2.5

### Metal specificity and analysis

**Fig 4:**
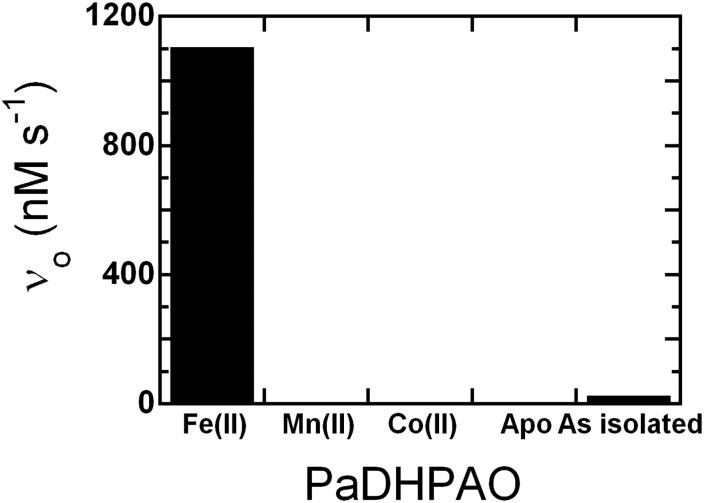
Specificity activities of PaDHPAO in the presence of various metal ions. The apoPaDHPAO was reconstituted with Fe(II), Mn(II), and Co(II) and assayed for activity. The results indicate that only purified and Fe(II)-reconstituted enzymes are active. Mn(II) and Co(II) cannot substitute Fe(II) for the activity requirement.

To explore whether PaDHPAO can use other metal ions as cofactors for its catalytic reaction, reconstitution of apoPaDHPAO with either of the two transition metal ions, Mn(II) or Co(II), was performed. The activities of Mn(II)- and Co(II)-reconstituted apoPaDHPAO were assayed and compared to that of Fe(II)-reconstituted apoPaDHPAO. The results (Fig 4) showed that the activity of apoPaDHPAO cannot be restored by substitution with either Mn(II) or Co(II). In order to identify whether PaDHPAO can bind to Mn(II) and Co(II), dye-binding thermal shift assay [39] in which a melting temperature (T_m_) was measured in the presence of Mn(II) or Co(II), was carried out. The results show that these two metal ions can bind to apoPaDHPAO because the T_m_ value of apoPaDHPAO (53°C, details shown later) was changed to higher temperatures in both Mn(II)- and Co(II)-reconstituted PaDHPAO. The T_m_ value of Mn(II)-reconstituted PaDHPAO was determined as 62°C while two T_m_ values, 61 and 72°C, were measured for Co(II)-reconstituted PaDHPAO (S2 Fig). These results clearly suggest that Fe(II) is the native and mandatory metal ion for PaDHPAO. These data are also in contrast to other previously obtained results for Fe(II)-dependent DHPAO from *B*. *fuscum*, in which Mn(II) and Co(II) were able to replace Fe(II) and provide similar or higher activities relative to the Fe(II)-native enzyme [28, 33]. Although a multiple sequence alignment suggests that PaDHPAO likely belongs to the mononuclear non-heme metal-containing 2-His-1-carboxylate facial triad enzyme superfamily (S1 Fig) and that it can also bind to Mn(II) and Co(II), unlike some enzymes in the same family, PaDHPAO specifically requires Fe(II) for its activity.

The mole ratios of Fe(II):enzyme in three different PaDHPAO samples −purified enzyme, apoenzyme, and Fe(II)-reconstituted enzymes− were analyzed by ICP-MS, MP-AES and ICP-OES. The results indicated that the mole ratio of Fe(II) to each enzyme form is 0.1:1, 0.05:1, and 0.71:1, respectively. The data imply that one PaDHPAO subunit can bind to approximately 1 atom of Fe(II) which is similar to the value of metal binding of DHPAOs from other sources (see Table 1). A mole ratio (0.1:1) of Fe(II) for the purified enzyme indicates that Fe(II) was lost significantly during the purification process. Therefore, the activation process of PaDHPAO by incubating the enzyme with a mixture of 10 mM DTT and 0.5 mM Fe(NH_4_)_2_(SO_4_)_2_ (see next results) is necessary before obtaining the active enzyme.

### PaDHPAO activation by redox agents

Various reducing agents including DTT, ascorbic acid and Fe(NH_4_)_2_(SO_4_)_2_ were examined for their ability to enhance enzymatic activity. The enzyme solution was incubated with these reagents at 25°C for 1 hour. The activity was assayed by adding 5 nM of the activated PaDHPAO into 50 mM sodium phosphate buffer, pH 7.5 and the reaction was started by adding 150 μM DHPA. The control experiment without enzyme was also carried out and did not result in any significant background of chemical oxidation. The measured specific activity of the enzyme after activation with the reducing reagents are shown in column 2 of Table 3. Activation with DTT alone did not improve the specific activity as compared to the non-activated enzyme, while a mixture of 10 mM DTT and 0.5 mM Fe(NH_4_)_2_(SO_4_)_2_ provides the highest enhancement of specific activity (54 U/mg, about 1.2 and 200 fold relative to the enzyme incubated with or without Fe(NH_4_)_2_(SO_4_)_2_ alone, respectively). Activation with ascorbic acid could also enhance the specific activity but the maximal activity was ~28% less than that of the enzyme activated with the mixture of DTT and Fe(NH_4_)_2_(SO_4_)_2_. This property of activation by ascorbic acid alone is different from DHPAO from *B*. *fuscum* in which the activity was regained to maximal levels when activated with ascorbic acid [33]. The ability to enhance specific activity of PaDHPAO was in the order of: mixture of DTT and Fe(NH_4_)_2_(SO_4_)_2_> Fe(NH_4_)_2_(SO_4_)_2_> ascorbic acid> DTT. Subsequently, activation of PaDHPAO was carried out by incubation with a mixture of 10 mM DTT and 0.5 mM Fe(NH_4_)_2_(SO_4_)_2_ before the enzyme was used in all experiments. The similar enzyme activation protocol using Fe(II) and a reducing agent to enhance specific activity was also found in 2,3-dihydroxyphenylpropionate 1,2-dioxygenase (MhpB) from *E*. *coli* in which addition of Fe(NH_4_)_2_(SO_4_)_2_ (0.05–10 mM) and ascorbic acid (1 mM) is mandatorily required for regaining a maximum specific activity of 48 U/mg protein [46].

**Table 3:** Specific activity of PaDHPAO after purification and incubation in various reducing agents.

Reducing agents used for enzyme activation	Specific activity (U/mg)
None	0.26 ± 0.03
10 mM DTT	0.26 ± 0.02
0.5 mM Ascorbic acid	14.3 ± 0.2
0.5 mM Fe(NH_4_)_2_(SO_4_)_2_	44 ± 2
Mixture of 10 mM DTT and 0.5 mM Fe(NH_4_)_2_(SO_4_)_2_	54 ± 3

### Preservation of the activated PaDHPAO activity

**Fig 5:**
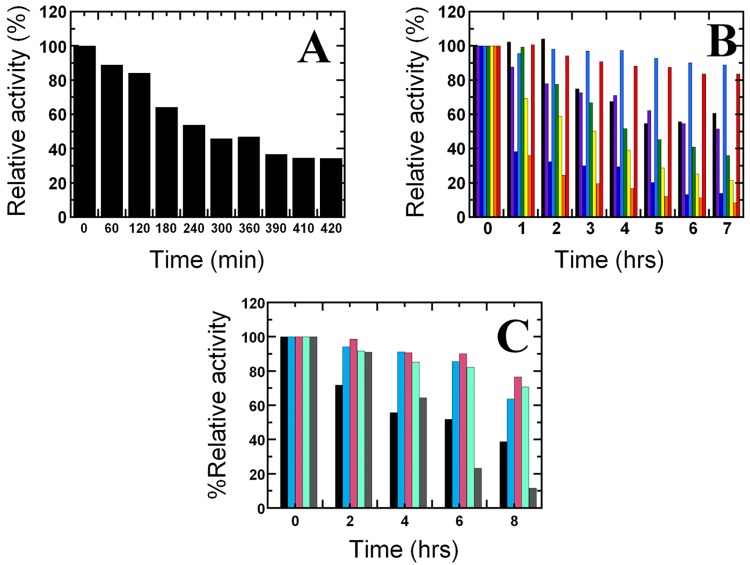
Preservation of the activated PaDHPAO activity. (A) Percentage of relative activity of the activated PaDHPAO decreased after the solution was kept on ice for 7–8 hours in 50 mM potassium phosphate buffer, pH 7.0. (B) Percentage of relative activity of the activated PaDHPAO in 50 mM potassium phosphate buffer, pH 7.0 containing different agents: (1) without any agents *(black)*, (2) 0.5 mM Fe(NH_4_)_2_(SO_4_)_2_ *(purple)*, (3) 1 mM DTT *(dark blue)*, (4) 0.5 mM ascorbic acid *(light blue)*, (5) 0.5 mM ascorbic acid and 1 mM DTT *(green)*, (6) 0.5 mM ascorbic acid, 1 mM DTT and 0.5 mM Fe(NH_4_)_2_(SO_4_)_2_ *(yellow)*, (7) 1 mM DTT and 0.5 mM Fe(NH_4_)_2_(SO_4_)_2_ *(orange)*, and (8) 0.5 mM ascorbic acid and 0.5 mM Fe(NH_4_)_2_(SO_4_)_2_ *(red)*. (C) Percentage of relative activity of the activated PaDHPAO in 50 mM potassium phosphate buffer, pH 7.0 containing different agents: (1) without any agents *(black)*, (2) 0.5 mM ascorbic acid *(cyan)*, (3) 1%(w/v) catalase *(pink)*, (4) 50 U/mL superoxide dismutase *(turquoise)* and (5) 1%(w/v) catalase and 50 U/mL superoxide dismutase *(brown)*. The results indicate that the highest level of % relative activity (around 90% up to ~ 6 hours) of the activated PaDHPAO can be achieved in the presence of 0.5 mM ascorbic acid *(cyan)*,catalase *(pink)* or superoxide dismutase *(turquoise)*).

The previous results as mentioned above indicated that the addition of 10 mM DTT and 0.5 mM Fe(NH_4_)_2_(SO_4_)_2_ is the best condition for PaDHPAO activation. However, we found that the % relative activity of the activated enzyme decreased to only 65% after 2 hours and 35% after 7 hours even when the enzyme was kept on ice (Fig 5A). This might be due to the oxidative degradation of enzyme which is caused by reactive oxygen species (ROS) that was generated in the presence of Fe(II) and DTT [47]. Therefore, in order to maintain activity of the activated enzyme, different mixtures of reducing agents including DTT, ascorbic acid, Fe(NH_4_)_2_(SO_4_)_2_ and ROS scavenging enzymes (catalase and superoxide dismutase) were tested to identify the reagent that could prolong the active Fe(II) state of the activated enzyme kept on ice. After activation by 10 mM DTT and 0.5 mM Fe(NH_4_)_2_(SO_4_)_2_, the activated enzyme solution was then exchanged into buffer with different reagents added (Materials and Methods) and then kept on ice. The activity of each enzyme sample (40 nM) was assayed at various storage times on ice (0 to 7 or 8 hours). The activity assay was carried out in 50 mM sodium phosphate buffer, pH 7.5 containing 390 μM DHPA. The control reaction without enzyme was also performed. The % relative activity of the enzyme under each of the conditions at different storage times was then compared. The results shown in Fig 5B indicate that the % relative activity of the activated enzyme in the presence of ascorbic acid (light blue) remained constant at almost 100% for up to 4 hours and only slightly decreased to 90% afterwards. Other conditions that were able to maintain high activity levels were those with Fe(NH_4_)_2_(SO_4_)_2_ and ascorbic acid added; however activities decreased after 1 hour (red color bars) (Fig 5B). The addition of catalase (pink) or superoxide dismutase (turquoise) can also preserve high level of enzyme activity similar to the addition of ascorbic acid (Fig 5C). Surprisingly, in the presence of both catalase and superoxide dismutase (brown), the enzyme activity was maintained at ~100% up to ~2 hours and then the activity rapidly decreased to be less than the level of only one enzyme usage (Fig 5C). The data suggest that the activity of the activated enzyme kept on ice can be preserved most efficiently with addition of either ascorbic acid, catalase or superoxide dismutase.

### Inactivation of PaDHPAO

To further investigate the influence of various metal ions and redox active reagents on the activity of PaDHPAO, the enzyme was incubated with the compounds shown in Table 4 at 4°C for either 10 min or 3.5 h. The percentage of activity remaining relative to the control of untreated PaDHPAO was measured over time. Similar to Fe(II)-dependent DHPAO from *B*. *fuscum* [28, 33], treatment with H_2_O_2_ and FeCl_3_ caused a rapid decrease of PaDHPAO activity. The inactivation by H_2_O_2_ is possibly due to the oxidation of Fe^2+^ to Fe^3+^. These data agree with our finding that Fe^2+^ is an essential catalytic cofactor. Fe^3+^ can also completely decrease the enzyme activity, possibly due to its oxidation power or to its binding to the enzyme active site and competing off Fe^2+^. The presence of 1 mM DTT also could not preserve the activity as the activity decreased by ~80% within 10 min and almost 100% within 3.5 h. A large decrease of PaDHPAO activity in the presence of any of H_2_O_2_, Fe^3+^ and DTT could be due to the nonenzymatic damage by hydroxyl radicals generated from a Fenton reaction [47]. However, this radical damage can be prevented by the addition of either ascorbic acid or an ROS scavenging enzyme (catalase or superoxide dismutase) (see results in Fig 5B and 5C). Divalent cations such as Mn^2+^ and Mg^2+^ had little effect on the activity (~80% remaining activity) while Zn^2+^ decreased the activity by ~40% after 3.5 h. This may be due to the replacement of the native Fe^2+^ cofactor by Zn^2+^ because Zn^2+^ can bind reasonably well to the Fe^2+^-dependent enzyme [33]. Anions such as F^-^ and N_3_^-^ had no effect on the PaDHPAO activity. As the activity was decreased only ~20% in the presence of 0.5 mM EDTA, the results suggest that the chelating agent at this concentration does not remove Fe^2+^ from the enzyme active site.

**Table 4:** Inactivation of PaDHPAO by metal ions and redox active reagents.

Reagents		%Activity remaining after incubating time at 4°C	
		10 min	3.5 h
Control	Untreated	98 ± 2	88 ± 2
Redox active reagents	5 mM NaF	89 ± 2	96 ± 7
	5 mM NaN_3_	84.7 ± 0.1	86 ± 1
	5 mM Na_2_SO_4_	81 ± 4	74 ± 4
	0.5 mM EDTA	75 ± 4	80 ± 1
	1 mM DTT	20 ± 3	11.1 ± 0.2
	1 mM H_2_O_2_	0.1 ± 0.1	0.2 ± 0.1
Metal ions	1 mM Fe(NH_4_)_2_(SO_4_)_2_	91.5 ± 0.1	90 ± 3
	5 mM MgCl_2_	81.6 ± 0.3	89 ± 3
	5 mM MnCl_2_	80 ± 8	78 ± 1
	5 mM ZnCl_2_	69 ± 5	62 ± 4
	1 mM FeCl_3_	0.2 ± 0.1	0.1 ± 0.1

### Characterization of the ring-cleavage product

**Fig 6:**
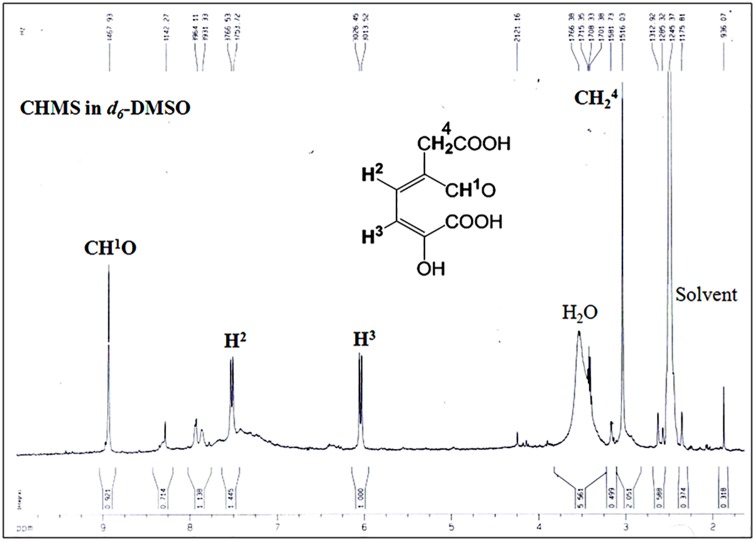
^1^H-NMR spectrum of the product from the PaDHAPO ring cleavage reaction. The product was isolated from the PaDHPAO reaction by ultrafiltration as described in the Methods section. The filtrate was lyophilized and resuspended in *d*_*6*_- DMSO.

The extradiol ring-cleavage product, 5-carboxymethyl-2-hydroxymuconate semialdehyde (CHMS), from the PaDHPAO reaction was analyzed by ^1^H-NMR. The NMR spectrum in Fig 6 shows a singlet at 8.93 ppm and two doublets centered at 6.04 (*J* = 15 Hz) and 7.52 ppm (*J* = 15 Hz). The singlet peak at 8.93 ppm is consistent with an aliphatic aldehyde proton (O = CH), suggesting that the ring-cleavage product is an extra-diol, while the other two doublet signals at 7.52 ppm and 6.04 ppm can be correlated with olefinic protons assigned as H^2^ and H^3^ (C = C**H**^**2**^-C**H**^**3**^ = C), respectively. A singlet peak at 3.1 ppm corresponds to the methylene protons (-C**H**_**2**_-COOH). The NMR spectrum indicates that the ring cleavage occurs at carbons 2 and 3.

**Fig 7:**
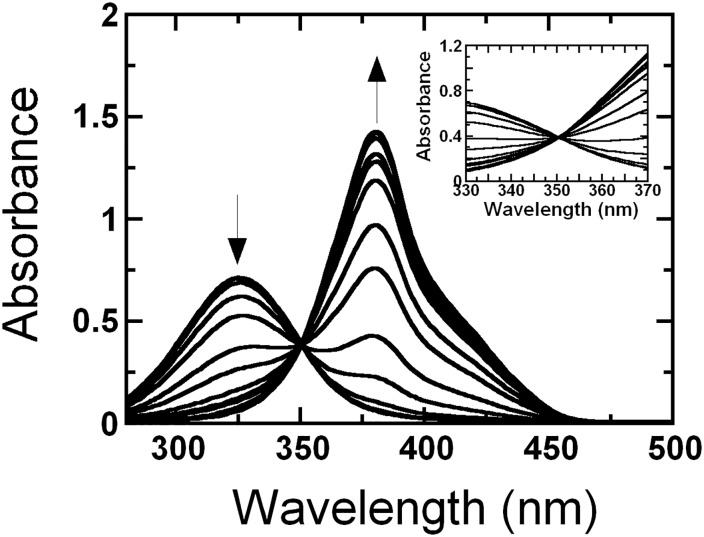
Absorbance spectra of the ring-cleavage product CHMS over a pH range of 4.5–12.0. Equal amounts of CHMS (30 μM) were added into 1-cm quartz cuvettes containing buffers at various pHs (pH 4.5–12.0). The buffers used in this experiment to maintain various pH values were 100 mM acetate/acetic acid (pH 4.5–5.5), 100 mM sodium phosphate (pH 6.0–7.5), 100 mM Tris-HCl (pH 8.0–8.5), 100 mM glycine adjusted with NaOH (pH 9.0–10.0), and 100 mM sodium phosphate (pH 10.5–12.0). Changes of absorbance at 325 and 380 nm from low to high pH are indicated by the direction of the arrows. A clear isosbestic point at 350 nm of the product spectra during pH change from pH 4.5–12.0 was observed (inset). The absorption coefficients at 380 and 350 nm of CHMS are 32.23 and 13.3 mM^-1^cm^-1^, respectively.

The influence of pH on the absorption spectra of the ring-cleavage product is shown in Fig 7. A clear isosbestic point at 350 nm is observed throughout a pH range of 4.5 to 12.0. While the absorbance at longer wavelengths such as at 380 nm increased upon raising the pH, the absorbance at shorter wavelengths such as 325 nm decreased. The absorption coefficient of the product at the maximum (380 nm) and isosbestic (350 nm) points were calculated to be 32.23 and 13.3 mM^-1^cm^-1^, respectively. The absorption characteristics of the PaDHPAO product are similar to those of the product from DHPAO isolated from *A*. *globiformis* CM-2 [24]. For calculating initial rates and specific activity at various pHs, the absorption coefficient value at 350 nm of 13.3 mM^-1^cm^-1^ was used in order to avoid any influence from pH variation.

### Substrate specificity

The specificity of PaDHPAO toward various catecholic compounds was investigated and the results are shown in Table 5. DHPAO catalyzes the extradiol ring cleavage of DHPA. It can also catalyze the cleavage of other catechol derivatives but with significantly lower activity. The *k*_cat_ value of the enzyme with 3,4-dihydroxyphenylpropionate (DHPP), an analog of DHPA which contains an additional methylene group between the carboxylate and catechol ring, is ~40 fold lower than that for the enzyme with DHPA. When the substrate side-chain is shorter, as is the case for the reaction with 3,4-dihydroxybenzoate (DHB), the activity is ~50,000 fold lower. These results are similar to the results observed for DHPAO from *P*. *ovalis* and *B*. *stearothermophilus* [19, 36]. Other compounds which contain only a single hydroxyl group such as 4-hydroxyphenylacetate, 4-hydroxybenzoate, 4-hydroxyphenylpropionate, 4-hydroxy-3-methoxyphenylacetate cannot be used as substrates, indicating that the substrates for PaDHPAO catalysis require two adjacent hydroxyl groups. According to studies by Lipscomb and co-workers, the catecholic moiety is required for coordination with the metal sphere [15, 16]. Based on the X-ray structures with various forms of intermediates trapped, it was suggested that one of the two hydroxyl groups of the catecholic substrate could be deprotonated to stabilize the coordination of the sphere complex which prompts the molecular oxygen to react with Fe(II) to readily generate a diradical intermediate of substrate-Fe(II)-superoxide complex. A radical pair derived from a catecholic substrate and Fe(II)-superoxide is collapsed to form an alkylperoxo intermediate, which then proceeds to form *gem*-diol, epoxide, and lactone intermediates before the ring breakage occurs [15, 16, 48].

**Table 5:** Percentage of relative activity of alternative substrates catalyzed by PaDHPAO.

Substrate	λ_max_ of product (nm)	%Relative activity
3,4-Dihydroxyphenylacetate	380	100
3,4-Dihydroxyphenylpropionate	380	2.6
3,4-Dihydroxybenzoate	355	0.002
4-Hydroxyphenylacetate	nd.	nd.
4-Hydroxybenzoate	nd.	nd.
4-Hydroxyphenylpropionate	nd.	nd.
4-Hydroxy-3-methoxyphenylacetate	nd.	nd.
4-Nitrocatechol	nd.	nd.

nd., not detectable

### Steady-state kinetics of PaDHPAO

**Fig 8:**
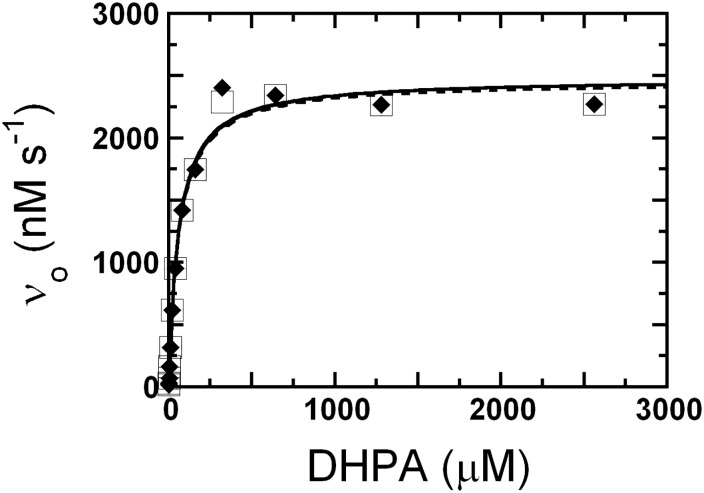
Steady-state kinetics of PaDHPAO prepared in buffers in the absence or presence of ascorbic acid. The assay reactions containing 39 nM PaDHPAO and various concentrations of DHPA (0.5–2560 μM) were carried out in air-saturated (oxygen concentration ~260 μM) 50 mM sodium phosphate buffer at pH 7.5 and 25°C and the reactions were monitored by the absorption increase at 380 nm. Plots of the initial rates of PaDHPAO prepared in buffers in the presence (a solid line with filled diamonds) or absence (a dashed line with empty squares) of ascorbic acid *versus* concentration of DHPA are shown. All kinetic parameters of both conditions are summarized in Table 6.

The steady-state kinetics of the activated PaDHPAO (39 nM) prepared in buffers with or without ascorbic acid was investigated using various concentrations of DHPA (0.5–2560 μM). The assay reactions were carried out in air-saturated (oxygen concentration ~260 μM) 50 mM sodium phosphate buffer at pH 7.5 and 25°C and the absorption at 380 nm was monitored. Plots of the initial rates of PaDHPAO prepared in buffers with or without ascorbic acid *versus* concentrations of DHPA are shown in Fig 8. All kinetics parameters obtained from the plots are summarized in Table 6. The results show that all kinetics parameters of the reaction of PaDHPAO prepared in buffers with or without ascorbic acid are similar; *k*_cat_, 62–63 s^-1^; *K*_m_ for DHPA, 58–67 μM; and *k*_cat_/*K*_m_, ~1 μM^-1^s^-1^ (Table 6). Because the reaction of PaDHPAO prepared in a buffer without ascorbic acid was performed within 2 hours, most of the enzyme was active (Fig 5B) with a turnover number similar to the reaction of PaDHPAO prepared in the buffer containing ascorbic acid.

**Table 6:** Summary of biochemical and kinetic properties of PaDHPAO.

Properties			Value
**Biochemical properties**	Metal content	Fe(II) (mole/subunit)	0.71
	Molecular weight (kDa)	Subunit molecular weight	30
		Native molecular weight	112.2
	Substrate specificity (%relative activity)	DHPA	100
		DHPP	2.6
		DHB	0.002
	Optimum pH		7.5
	Optimum temperature (°C)		55
	Transition temperature (°C)		35
	Melting temperature (°C)		50–55
**Kinetic properties**	In the absence of ascorbic acid (assayed within 2 hour)	*k*_cat_ (s^-1^)	62.4
		*K*_m, DHPA_ (μM)	67 ± 7
		*k*_cat_/*K*_m, DHPA_ (μM^-1^s^-1^)	0.9
	In the presence of ascorbic acid	*k*_cat_ (s^-1^)	63.5
		*K*_m, DHPA_ (μM)	58 ± 8
		*k*_cat_/*K*_m, DHPA_ (μM^-1^s^-1^)	1.1

The *k*_cat_ of PaDHPAO (62–63 s^-1^) is the highest among the values of all DHPAOs previously reported including Fe(II)-dependent DHPAO from *B*. *fuscum* (HPCD) [20, 49], Mn(II)-dependent DHPAOs from *A*. *globiformis* CM-2 (MndD) [24] and *B*. *brevis* [21] (Table 1). It is interesting to note that in addition to PaDHPAO, two other DHPAOs (from *K*. *pneumoniae* and *E*. *coli* C) which are members of Cluster 1 (Fig 2) are also enzymes with high specific activities (Table 1). We would like to propose that DHPAO members of Cluster 1 are dioxygenases with fast turnovers. The results reported herein can thus be used as a representative to understand the catalytic properties of the enzymes in this cluster.

### Effects of temperature and pH on the catalysis of PaDHPAO

**Fig 9:**
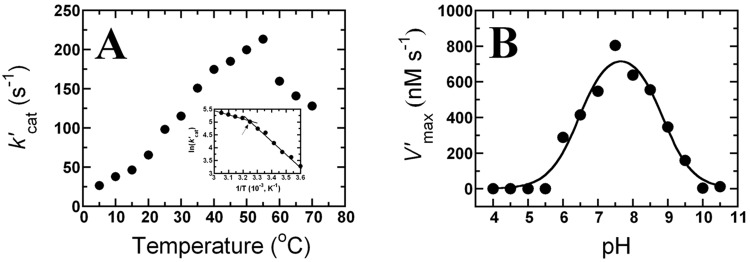
Effects of temperature and pH on the catalysis of PaDHPAO. (A) To evaluate the effects of temperature on activity, the *k’*_cat_ values were measured at 5–70°C and plotted as a function of temperature. The inset in A is the Arrhenius plot of ln(*k’*_cat_) versus the reciprocal of the absolute temperature (5–55°C). The data indicate a transition temperature at 35°C (arrow). (B) To evaluate the effect of pH on activity, the apparent maximum velocities (*V’*_max_) of the PaDHPAO reaction were measured under various pH conditions (4.0–10.5). A plot of the pH-rate profile indicates an optimal pH at 7.5 with two p*K*_a_ values of 6.5 ± 0.1 and 8.9 ± 0.1, respectively.

Evaluation of the effect of temperature on the activity of PaDHPAO was performed at a saturating concentration of DHPA (400 μM). The *k’*_cat_ values were measured over a temperature range of 5–70°C. The results indicated that the catalytic activity of PaDHPAO increased upon increasing the temperature up to 55°C and then decreased at higher temperatures (60–70°C) (Fig 9A). These data agree with the reaction of DHPAO from *B*. *stearothermophilus* which showed a maximum activity at 52°C [36]. The Arrhenius plot of ln(*k’*_cat_) *versus* reciprocal values of absolute temperature (5–55°C) showed a pattern of two straight lines with a transition temperature at 35°C (Inset of Fig 9A). The slope of each straight line can be fitted to the Arrhenius equation and the activation energies (*E*_a_) below (5–35°C) and above (35–55°C) were determined as 42 and 14 kJ/mol, respectively. The data suggest that the rate-limiting steps of PaDHPAO reaction at temperatures above and below 35°C are different. Similar results were previously found for DHPAO from *B*. *stearothermophilus* in which the temperature break point was at 32°C [36]. The analysis of PaDHPAO using the dye-binding thermal shift assay technique showed that the T_m_ of PaDHPAO is 50–55°C (S3 Fig). The data suggest that PaDHPAO is a thermostable enzyme that could be useful for future applications in biotechnology.

To evaluate the effects of pH on the PaDHPAO reaction, the enzyme activity was measured at various pH conditions (4.0–10.5). The apparent maximum velocities (*V’*_max_) obtained from the reaction catalyzed by PaDHPAO were plotted as a function of pH. The pH-rate profile showed a bell-shaped curve (Fig 9B) which indicated an optimal pH for the PaDHPAO reaction of 7.5 which is the condition that has been used throughout this study. This result is in contrast to the DHPAOs from *P*. *ovalis* [18], *B*. *stearothermophilus* [36], and *B*. *fuscum* [49] in which the optimal pH values were found to be 8.0, 8.2, and 8.5, respectively. When the bell-shaped curve was fitted by eq (1), two p*K*_a_ values were determined as 6.5 ± 0.1 and 8.9 ± 0.1, respectively (Fig 9B). The results suggest that a functional group with a p*K*_a_ of ~6.5 should be deprotonated, while the other functional group with a p*K*_a_ of ~8.9 should be protonated in order to obtain the maximum rate for the PaDHPAO-catalyzed reaction using DHPA as the substrate. These two p*K*_a_ values of PaDHPAO reaction are similar to the p*K*_a_ values of 6.4 and 8.8 found in the pH-rate profile of the reaction of *E*. *coli* 2,3-dihydroxyphenylpropionate 1,2-dioxygenase (MhpB). Studies in the previous report indicate that the p*K*_a_ values of 6.4 and 8.8 likely correspond to the deprotonated and protonated form of His179 and His115 that are required to act as a general base and acid in the reaction of MhpB, respectively [50].

## Conclusion

3,4-Dihydroxyphenylacetate 2,3 dioxygenase from *Pseudomonas aeruginosa* (PaDHPAO) was overexpressed, purified and characterized for its enzymatic properties as summarized in Table 6. The results showed that PaDHPAO belongs to an enzyme cluster that is largely unexplored. Overexpression of the enzyme in *E*. *coli* BL21 (DE3) yielded ~221 mg per 3.2 liters of cell culture. Metal analysis and activity assays with enzyme reconstituted with various metals indicated that Fe(II) is the native cofactor of PaDHPAO and that the enzyme contains 1 Fe per subunit. The loss of PaDHPAO activity during purification could be restored by adding a mixture of Fe(NH_4_)_2_(SO_4_)_2_ and DTT. Ascorbic acid or ROS scavenging enzyme (catalase or superoxide dismutase) can be used as a preservative agent for maintaining the activity of activated PaDHPAO. Analysis of the enzyme product by ^1^H-NMR has clearly identified PaDHPAO as an extradiol ring cleavage 2,3-dioxygenase. PaDHPAO is specific to catecholic substrates with vicinal hydroxyl groups at the 3 and 4 positions. Furthermore, a single methylene moiety between the carboxylate and phenyl group is required for the most optimal activity. The optimum temperature and pH for PaDHPAO catalysis is 55°C and pH 7.5, respectively. As PaDHPAO and the other two enzymes in Cluster 1 all have high specific activities, it is possible that DHPAO members of this cluster are also dioxygenases with fast turnovers. As most of the enzymes in this cluster are largely unexplored, the results reported herein are useful for establishing an understanding of the catalytic properties of these dioxygenases.

## Supporting information

S1 Fig A multiple sequence alignment of PaDHPAOs and other extradiol-ring cleavage dioxygenase in Cluster 1 and Cluster 6 (referred to Clusters in Fig 2 main text). (A) The amino acid sequence of PaDHPAO was aligned with multiple sequences of *Klebsiella pneumoniae* (Kp) and *Escherichia coli* C (Ec) DHPAO enzymes in Cluster 1 and APD from *Comamonas testosteroni* (Ct) in Cluster 6 (A). Three amino acid residues (highlighted in yellow color) of PaDHPAO (His12, His57, and Glu239) and other DHPAOs in Cluster 1 are well conserved when compared to the CtAPD active-site residues (His13, His62, and Glu251). The other two active-site residues (highlighted in cyan, Tyr129 and His195) are also conserved in PaDHPAO (Tyr125 and His186) and other DHPAOs in Cluster 1. These two residues are important for second sphere metal-coordination and were shown to be important for catalysis [42]. (B) The active site structure of CtAPD co-crystallized with 4-nitrocatechol (4NC) shows the involvement of all these conserved residues in metal coordination and substrate binding. As these residues are conserved in PaDHPAO, it is likely that the enzyme is a member of the 2-His-1-carboxylate enzyme superfamily similar to CtAPD.
(PDF)

S2 Fig Measurement of the binding of apoPaDHPAO with each metal candidate (Fe(II), Mn(II), and Co(II)) by a dye-binding thermal shift assay. Fluorescence changes of a SYPRO Orange dye (Invitrogen, USA) in the presence of apoPaDHPAO and each metal candidate (Fe(II), Mn(II) or Co(II)) (0.5 mM) were monitored using a real-time PCR machine with temperature increase from 25–95°C with a constant increment of 1°C/min. The normalized (A) and derivative (B) curves of thermal shift showed that the melting temperature (T_m_) of PaDHPAO-Fe(II) complex (yellow) is ~53°C which is similar to that of apoPaDHPAO (red). The T_m_ values of DHPAO-Mn(II) (green) was measured as 62 while that of PaDHPAO-Co(II) (turquoise) was measured as and 61 and 72°C.
(PDF)

S3 Fig Thermal stability of PaDHPAO. Fluorescence changes of a SYPRO Orange dye (Invitrogen, USA) in the presence of three PaDHPAO samples including apoPaDHPAO, activated PaDHPAO, and activated PaDHPAO plus ascorbic acid were monitored using a real-time PCR machine with temperature increase from 25–95°C with a constant increment of 1°C/min. The normalized (A) and derivative (B) curves of thermal stability showed that the melting temperature (T_m_) of three PaDHPAO samples, apoPaDHPAO (yellow), activated PaDHPAO (cyan), and activated PaDHPAO plus ascorbic acid (dark blue), are in a similar temperature range of 50–55°C.
(PDF)
