# Supplementary material for: 3,4-Dihydroxyphenylacetate 2,3-dioxygenase from Pseudomonas aeruginosa: An Fe(II)-containing enzyme with fast turnover
Source: PLoS One. 2017 Feb 3;12(2):e0171135. doi: 10.1371/journal.pone.0171135 (PMC5291488; doi:10.1371/journal.pone.0171135)
Supplement: S1 Fig — (A) The amino acid sequence of PaDHPAO was aligned with multiple sequences of Klebsiella pneumoniae (Kp) and Escherichia coli C (Ec) DHPAO enzymes in Cluster 1 and APD from Comamonas testosteroni (Ct) in Cluster 6 (A). Three amino acid residues (highlighted in yellow color) of PaDHPAO (His12, His57, and Glu239) and other DHPAOs in Cluster 1 are well conserved when compared to the CtAPD active-site residues (His13, His62, and Glu251). The other two active-site residues (highlighted in cyan, Tyr129 and His195) are also conserved in PaDHPAO (Tyr125 and His186) and other DHPAOs in Cluster 1. These two residues are important for second sphere metal-coordination and were shown to be important for catalysis [42]. (B) The active site structure of CtAPD co-crystallized with 4-nitrocatechol (4NC) shows the involvement of all these conserved residues in metal coordination and substrate binding. As these residues are conserved in PaDHPAO, it is likely that the enzyme is a member of the 2-His-1-carboxylate enzyme superfamily similar to CtAPD. (PDF) [file pone.0171135.s001.pdf]

Supporting information

(A)

```
CtAPD               MQGEIIAGFLAPHPPHLVYGENPPQNEPRSQGGWEVLRWAYERARERLDAMKPDVLLVHS    60
PaDHPAO             -MGKVALAAKITHVPSLYLSELPGPRHGCRQPAIDGHR----EIGRRCRELGVDTIVVFD    55
KpDHPAO             -MGKLALAAKTTHVPSMYLSELPGKNHGCRQGAIDGHK----EIGKRCRELGVDTFIVFD    55
EcDHPAO             -MGKLALAAKITHVPSMYLSELPGKNHGCRQGAIDGHK----EISKRCREMGVDTIIVFD    55
                      *::  .    * * :  .* *  ..   * . :  :    .  .*   :  *.::*..

CtAPD               PHWITSVGHHFLGVPELSGKSVDPIFPN---VFRYDFSLNVDVELAEACAEEGRKAGLVT    117
PaDHPAO             THWLVNAGYHINCAPHFEGLYTSNELPHFIANMEYGFP--GNPELGRILAEGCNALGVET    113
KpDHPAO             THWLVNSAYHINCADHFQGVYTSNELPHFIRDMTYDYD--GNPELGHLIADKTVKLGVRA    113
EcDHPAO             THWLVNSAYHINCADHFEGVYTSNELPHFIRDMTYNYE--GNPELGQLIADEALKLGVRA    113
                     **:.. .:*:  . .:.*  ..  :*:    : *.:    : **..  *:     *: :

CtAPD               KMMRNPKFRVDYGTITTLHLIRPQWDIPVVGISANNSPYYLNTKEGMSEMDVLGKATREA    177
PaDHPAO             LAHDATTLGPEYGTLVPMRYMNQDRHFKVVSVSALCTVHYLND------SARLGWAMRKA    167
KpDHPAO             KAHNIPSLKLDYGTLVPMRYMNADKHFKVVSISAFCTVHDFAD------SRKLGEAIRKA    167
EcDHPAO             KAHNIPSLKLEYGSVVPMRYMNEDKRFKVVSISAFCTVHDFAD------SRKLGERIVKA    167
                          .:  :**::. :: :. :  : **.:**  : : :           **    :*

CtAPD               IRK-TGRKAVLLASNTLSHWHFHEEPTIPEDMSKEYPATMAGYQWDIRMIELMRQGKTSE    236
PaDHPAO             VEEHYDGTVAFLASGSLSHRFAQ-NGQAPDFSDR-IWS-PFLEVLDHEVVQMWQEGRWAE    224
KpDHPAO             I-EKYDGTVAVLASGSLSHRFIE-DQRAEEGMNS-YTR-EFDHQMDERVVKLWREGKFKE    223
EcDHPAO             I-EQYDGTVAVLASGSLSHRFID-DQRAEEGMNS-YTR-EFDRQMDERVVKLWREGQFKE    223
                    : :  . ....***.:*** . . :    :  .            * .:::: ::*:  *

CtAPD               VFKLLPQFIDEAFAEVKSGAFTWMHAAMQYPELAAELFGYGTVIGTGNAVMEWDLRKAG-    295
PaDHPAO             FCGMLPEYASKGHGEGFMHDTAML----------LGALGWSAYDGK-----AEVVTPYFG    269
KpDHPAO             FCTMLPENAEYCYGEGNMHDTVML----------LGLLGWDKYDGK-----EWNLSPNCL    268
EcDHPAO             FCNMLPEYADYCYGEGNMHDTVML----------LGMLGWDKYDGK-----VWSLSPSYS    268
                    .  :**:  .  ..*      . :             :*:.   *.        :

CtAPD               ---LSMLGA------ADQKQRSAAVA------------          312
PaDHPAO             SSGTGQINAVFPVTAQDGSAIPAAQAGNPAGASCASRL          307
KpDHPAO             PASGTRPG------------------------------          276
EcDHPAO             QASWHRSG------------------------------          276
                           .
```

(B)

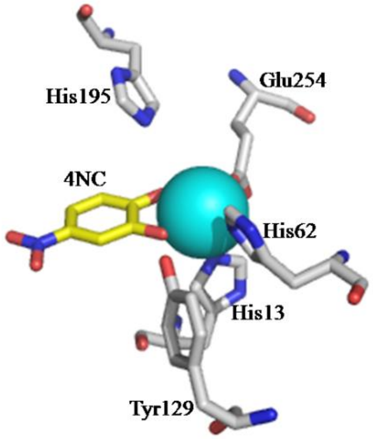

S1 Fig. A multiple sequence alignment of PaDHPAOs and other extradiol-ring cleavage dioxygenase in Cluster 1 and Cluster 6 (referred to Clusters in Fig. 2 main text). (A) The amino acid sequence of PaDHPAO was aligned with multiple sequences of *Klebsiella pneumoniae* (Kp) and *Escherichia coli* C (Ec) DHPAO enzymes in Cluster 1 and APD from *Comamonas testosteroni* (Ct) in Cluster 6 (A). Three amino acid residues (highlighted in yellow color) of PaDHPAO (His12, His57, and Glu239) and other DHPAOs in Cluster 1 are well conserved when compared to the CtAPD active-site residues (His13, His62, and Glu251). The other two active-site residues (highlighted in cyan, Tyr129 and His195) are also conserved in PaDHPAO (Tyr125 and His186) and other DHPAOs in Cluster 1. These two residues are important for second sphere metal-coordination and were shown to be important for catalysis [42]. (B) The active site structure of CtAPD co-crystallized with 4-nitrocatechol (4NC) shows the involvement of all these conserved residues in metal coordination and substrate binding. As these residues are conserved in PaDHPAO, it is likely that the enzyme is a member of the 2-His-1-carboxylate enzyme superfamily similar to CtAPD.
