# Supplementary material for: 3,4-Dihydroxyphenylacetate 2,3-dioxygenase from Pseudomonas aeruginosa: An Fe(II)-containing enzyme with fast turnover
Source: PLoS One. 2017 Feb 3;12(2):e0171135. doi: 10.1371/journal.pone.0171135 (PMC5291488; doi:10.1371/journal.pone.0171135)
Supplement: S2 Fig — Fluorescence changes of a SYPRO Orange dye (Invitrogen, USA) in the presence of apoPaDHPAO and each metal candidate (Fe(II), Mn(II) or Co(II)) (0.5 mM) were monitored using a real-time PCR machine with temperature increase from 25–95°C with a constant increment of 1°C/min. The normalized (A) and derivative (B) curves of thermal shift showed that the melting temperature (Tm) of PaDHPAO-Fe(II) complex (yellow) is ~53°C which is similar to that of apoPaDHPAO (red). The Tm values of DHPAO-Mn(II) (green) was measured as 62 while that of PaDHPAO-Co(II) (turquoise) was measured as and 61 and 72°C. (PDF) [file pone.0171135.s002.pdf]

Supporting information

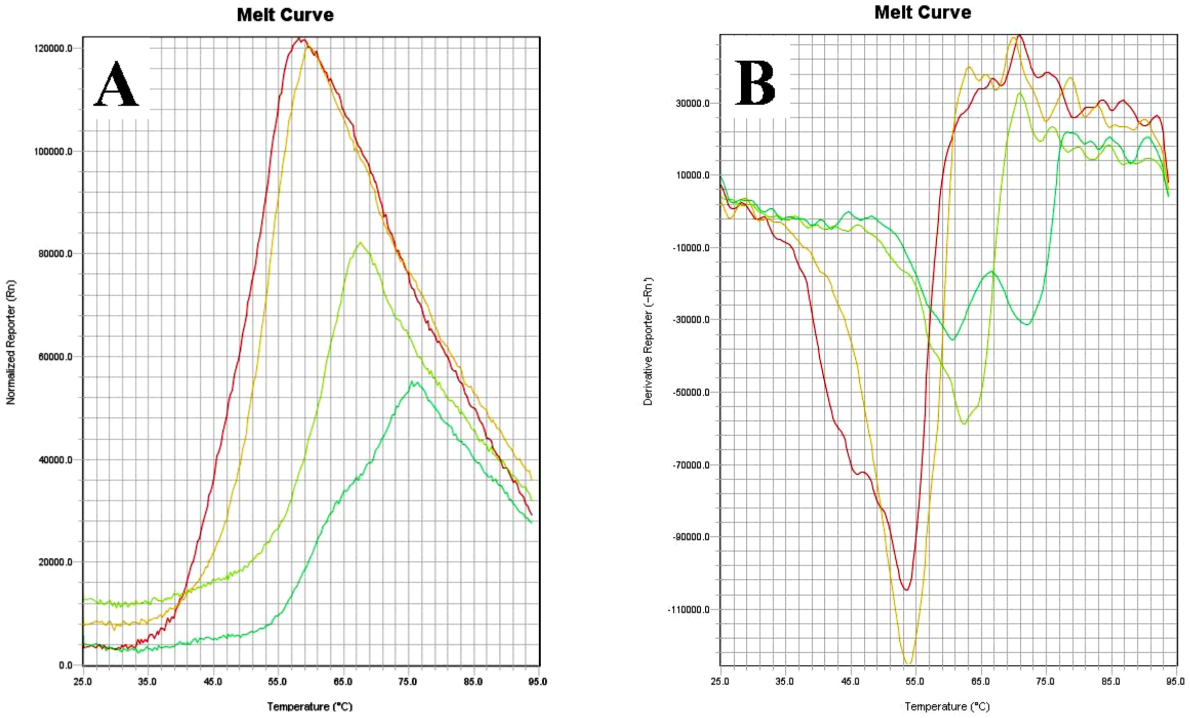

S2 Fig. Measurement of the binding of apoPaDHPAO with each metal candidate (Fe(II), Mn(II), and Co(II)) by a dye-binding thermal shift assay. Fluorescence changes of a SYPRO Orange dye (Invitrogen, USA) in the presence of apoPaDHPAO and each metal candidate (Fe(II), Mn(II) or Co(II)) (0.5 mM) were monitored using a real-time PCR machine with temperature increase from 25-95 ^{o}C with a constant increment of 1 ^{o}C/min. The normalized (A) and derivative (B) curves of thermal shift showed that the melting temperature (T_m) of PaDHPAO-Fe(II) complex (yellow) is ~53 ^{o}C which is similar to that of apoPaDHPAO (red). The T_m values of DHPAO-Mn(II) (green) was measured as 62 while that of PaDHPAO-Co(II) (turquoise) was measured as and 61 and 72 ^{o}C.
