# Supplementary material for: 3,4-Dihydroxyphenylacetate 2,3-dioxygenase from Pseudomonas aeruginosa: An Fe(II)-containing enzyme with fast turnover
Source: PLoS One. 2017 Feb 3;12(2):e0171135. doi: 10.1371/journal.pone.0171135 (PMC5291488; doi:10.1371/journal.pone.0171135)
Supplement: S3 Fig — Fluorescence changes of a SYPRO Orange dye (Invitrogen, USA) in the presence of three PaDHPAO samples including apoPaDHPAO, activated PaDHPAO, and activated PaDHPAO plus ascorbic acid were monitored using a real-time PCR machine with temperature increase from 25–95°C with a constant increment of 1°C/min. The normalized (A) and derivative (B) curves of thermal stability showed that the melting temperature (Tm) of three PaDHPAO samples, apoPaDHPAO (yellow), activated PaDHPAO (cyan), and activated PaDHPAO plus ascorbic acid (dark blue), are in a similar temperature range of 50–55°C. (PDF) [file pone.0171135.s003.pdf]

Supporting information

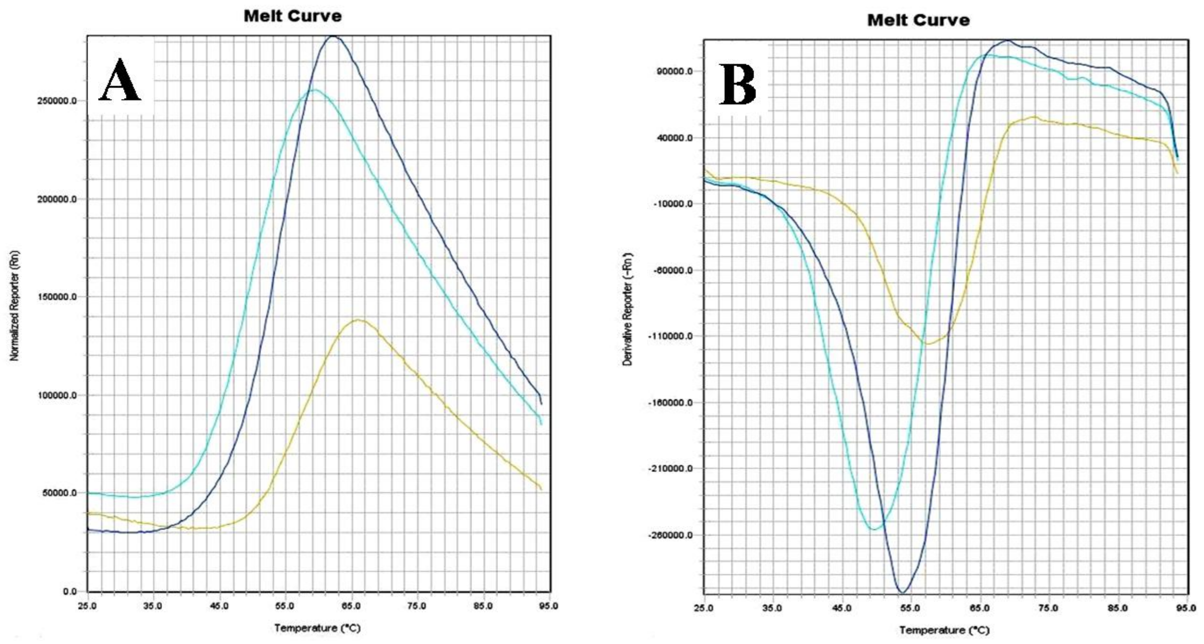

S3 Fig. Thermal stability of PaDHPAO. Fluorescence changes of a SYPRO Orange dye (Invitrogen, USA) in the presence of three PaDHPAO samples including apoPaDHPAO, activated PaDHPAO, and activated PaDHPAO plus ascorbic acid were monitored using a real-time PCR machine with temperature increase from 25-95 ^{o}C with a constant increment of 1 ^{o}C/min. The normalized (A) and derivative (B) curves of thermal stability showed that the melting temperature (T_m) of three PaDHPAO samples, apoPaDHPAO (yellow), activated PaDHPAO (cyan), and activated PaDHPAO plus ascorbic acid (dark blue), are in a similar temperature range of 50-55 ^{o}C.
